# Monitoring of mitochondrial oxygenation during perioperative blood loss

**DOI:** 10.1136/bcr-2020-237789

**Published:** 2021-01-19

**Authors:** Floor A Harms, Alexandra R M Brandt-Kerkhof, Egbert G Mik

**Affiliations:** 1 Laboratory for Experimental Anesthesiology, Department of Anesthesiology, Erasmus MC, Rotterdam, Zuid-Holland, The Netherlands; 2 Surgery, Erasmus MC, Rotterdam, Zuid-Holland, The Netherlands

**Keywords:** anaesthesia, haematology (incl blood transfusion), surgical oncology

## Abstract

One of the challenges in the management of acute blood loss is to differentiate whether blood transfusion is required or not. The sole use of haemoglobin values might lead to unnecessary transfusion in individual cases. The suggestion is that mitochondrial oxygen tension can be used as an additional monitoring technique to determine when blood transfusion is required. In this case report, we report mitochondrial oxygen measurements in a patient with perioperative blood loss requiring blood transfusion.

## Background

Goal-directed management of perioperative blood loss remains a major challenge for clinicians. Acute anaemia resulting in inadequate oxygen supply should be avoided at all times, but currently, no specific endpoint for personalised transfusion medicine is available. Perioperative insufficient oxygen delivery to tissues is an important determinant for postoperative complications such as stroke,^1^ declined cognitive function,^2^ kidney injury^3^ and cardiac ischaemia.^4^ Tissue oxygenation relies on adequate oxygen delivery, which is predominantly maintained by the arterial oxygen saturation, haemoglobin concentration and cardiac output. Transfusion of allogeneic erythrocyte concentrates plays an important role in treating acute anaemia for the prevention of tissue hypoxia. However, allogeneic blood transfusion itself is not without risks and is an independent risk factor for increased mortality and morbidity.^5^ One of the challenges in the management of anaemia is to determine whether blood transfusion is required or not. Current transfusion guidelines recommend haemoglobin levels as a trigger for red blood cell transfusion.^6 7^ These guidelines are based on mean data and thus incorporate safety margins, which might lead to unnecessary transfusion in individual cases. Additionally, more physiologically based transfusion triggers may enable optimisation and personalised treatment during the management of acute blood loss and may help in preventing transfusion-related complications like haemolytic reactions, transfusion-related acute lung injury, infections and transfusion-associated circulatory overload. In a recent study in haemodiluted pigs, mitochondrial oxygen tension (mitoPO_2_) was suggested as a useful parameter to distinguish whether blood transfusion is necessary or not.^8^ Given that the mitochondrion is the final destination of oxygen, it seems logical to use mitoPO_2_ as a measure for transfusion need. The mitoPO_2_ can be measured by the COMET (an acronym for Cellular Oxygen METabolism) measuring system (Photonics Healthcare, Utrecht, the Netherlands).^9^ The measurement is based on the principle of oxygen-dependent quenching of delayed fluorescence of protoporphyrin IX (PpIX).^10 11^ Application of the porphyrin precursor 5-aminolevulinic acid (5-ALA) on the skin induces PpIX in the mitochondria where it acts as a mitochondrially located oxygen-sensitive dye.^12^ After photoexcitation with a pulse of green light, PpIX emits delayed fluorescence of which the lifetime is inversely related to the amount of oxygen. The technique is non-invasive and can be safely used in humans.^13 14^ In this case report, we report the results of mitochondrial oxygen measurements in a patient with major intraoperative blood loss requiring blood transfusion.

## Case presentation

A 69-year-old man with a history of diabetes mellitus (type II), hypertension and dyslipidaemia and a recent diagnosis of metastatic ascending colon carcinoma (cT4N2M1) required extensive surgery and hyperthermic intraperitoneal chemotherapy (HIPEC). His medication included metformin, gliclazide, enalapril and simvastatin. Preoperative abdominal, respiratory and cardiac examination were unremarkable. Preoperative haemoglobin levels were 93.5 g/L.

Prior to induction of anaesthesia, an epidural catheter was placed, and epidural analgesia (ropivacaine/sufentanil) was given. For induction of anaesthesia, an intravenous bolus dose of propofol 110 mg followed by rocuronium 50 mg was administered, together with a continuous infusion of remifentanil 9 µg/kg/hour. Anaesthesia was maintained using sevoflurane. Directly after induction of anaesthesia, a continuous infusion of noradrenalin (0.40–0.60 µg/kg/min) was necessary to maintain normal blood pressure values (mean arterial pressure (MAP) >65 mm Hg).

During surgery, extensive peritoneal carcinomatosis was observed with a Peritoneal Carcinomatosis Index of 16.^15^ The surgeons performed a low anterior resection and an omentectomy. Thereafter, warm mitomycin C (chemotherapeutic agent) was rinsed abdominally for 1.5 hours via three inflow and two outflow catheters. Finally, a previously constructing ileostomy was reversed, and a terminal colostomy was placed.

**Figure 1:**
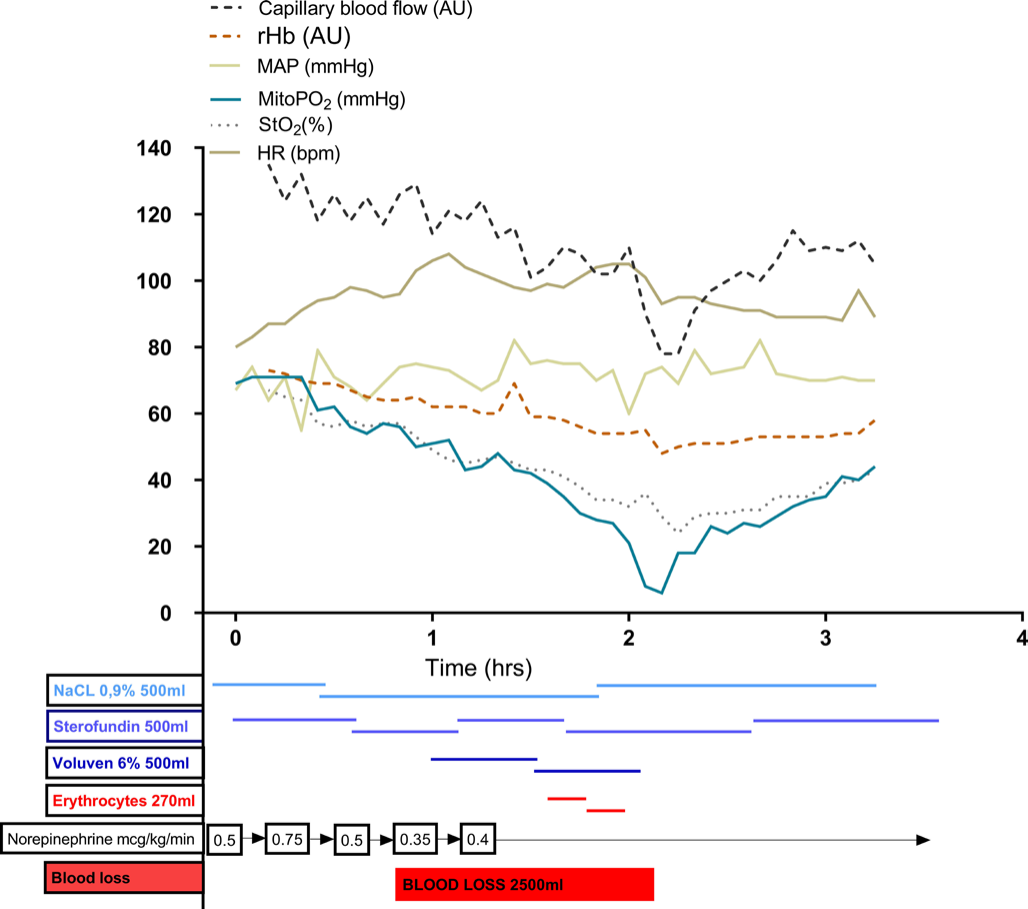
Haemodynamics and transfusion during surgery. HR, heart rate; MAP, mean arterial pressure; MitoPO_2_, mitochondrial oxygen tension; rHb, recombinant haemoglobin; StO_2_, tissue oxygen saturation.

After 1 hour of coma, during the low anterior resection, an acute rapid blood loss of 2500 mL occurred. Resuscitation of blood loss was initially done using crystalloids and colloids (figure 1). Haemodynamic parameters were kept stable, without the need for extra vasoactive medication. Blood transfusion was started after haemoglobin levels dropped below 88.6 g/L.

## Investigations

In addition to the standard intraoperative monitoring consisting of invasive blood pressure measurements, peripheral oxygen saturation, 5-lead electrocardiography and temperature measurements, two additional monitoring systems were used during this operation: the COMET for monitoring mitochondrial oxygenation and the oxygen to see (O2C) (LEA Medizintechnik, Giessen, Germany) for monitoring microcirculatory blood flow velocity, tissue oxygen saturation (StO_2_) and relative amount of recombinant haemoglobin (rHb) in the skin.^16^

For the COMET measurements, a 5-ALA patch was applied the evening before surgery. Directly after the induction of anaesthesia, the 5-ALA patch was removed, and the skin sensor of the COMET was fixated to the chest. COMET measurement was performed during surgery with an interval of one measurement per minute. The O2C probe was placed on the skin of the sternum next to the COMET measurement probe providing semicontinuous readings.

## Outcome and follow-up

The mitoPO_2_ value started around 70 mm Hg and slowly declined in the first hour, parallel to StO_2_, to reach values around 50–60 mm Hg. After approximately 1 hour of operation time, a sudden blood loss of 2.5 L occurred. Initially, adequate haemodynamic status was ensured by infusion of crystalloids and colloids resulting in haemodilution and acute anaemia. Due to anaemia, oxygen delivery to the tissues decreased, accompanied by declining microvascular and mitochondrial parameters, while other parameters such as MAP, StO_2_, rHb and lactate levels did not change during the ongoing blood loss. Heart rate and capillary blood flow did show a response to the bleeding but at a later stage than mitoPO_2_. Directly after resuscitation with red blood cells, a rapid increase of mitoPO_2_ was observed, with mitoPO_2_ values restored from below 10 mm Hg to up to 40 mm Hg (figure 1).

After surgery, the patient was transferred to the intensive care unit (ICU), and discharge from the ICU to a surgical ward was possible after 2 days. The stay in the surgical ward was complicated by a paracolic fluid collection, which was surgically drained. After a further trouble-free recuperation, the patient was released from the hospital 15 days after surgery. Eight months later, a CT scan diagnosed extensive recurrence of disease, eliminating curative treatment options. Unfortunately, the patient died 1 year later from the consequences of his illness.

## Discussion

In this case report, we show mitoPO_2_ use during acute perioperative blood loss and propose mitoPO_2_ as an additional monitoring parameter for perioperative transfusion management.

In the current literature, there is controversy regarding the transfusion of blood components in oncological surgery. As early as in the 1980s, the effect of blood transfusion on malignancy recurrence rate was noted.^17^ These findings were based on simple bivariate correlation without the adjustment of confounders. The correlation between blood transfusion and malignant recurrence rate became less obvious with the introduction of new statistical techniques whereby confounding factors were included in the analysis.^18 19^ Although the negative effect of blood components does not appear to apply in all cancers types, it is not the case in patients with peritoneal colorectal carcinomatosis undergoing cytoreductive surgery and HIPEC. Two recently published articles both showed an independent relationship between perioperative blood transfusion and survival rate, especially in patients with high-grade mucinous neoplasms.^18 19^ These findings underline the importance of blood-sparing protocols during cytoreductive surgery and HIPEC. In this case report, the potential value of perioperative monitoring of the mitochondrial oxygenation in the decision as to whether or not to transfuse a patient is shown. The mitochondria are the largest oxygen consumers within the cell. Therefore, mitoPO_2_ reflects the oxygen balance between oxygen supply and oxygen demand.^20^ Oxygen supply is dependent not only on the amount of haemoglobin but also on microvascular blood flow, the haemoglobin dissociation characteristics, the level of oxygen saturation of haemoglobin and the diffusion barriers between red blood cells and the tissue cells.^21^ Because so many factors are involved in maintaining an adequate tissue oxygenation, it doesn’t seem wise to use only haemoglobin levels in the decision of transfusing red blood cells. Therefore, we suggest to use mitoPO_2_ and microvascular flow measurements in combination with point-of-care haemoglobin and standard operative measurements, such as blood pressure, heart rate and pulse oximetry, in the decision-making process for blood transfusion. The main goal is to reduce the number of blood transfusions required during oncological surgery, particularly during cytoreductive surgery and HIPEC and thereby improve the long-term outcome of the patients. The added value of the mitochondrial oxygenation measurements during acute perioperative blood loss must be demonstrated in future studies.

**Learning points**

- Transfusion is not without risk; a more individualised threshold for determining blood transfusion is needed.
- Monitoring of oxygenation at the mitochondrial level is clinically possible by using the delayed fluorescence of protoporphyrin IX.
- Mitochondrial oxygenation monitoring provides a new tool for research in resuscitation and transfusion medicine.
